# Exogeneous metal ions as therapeutic agents in cardiovascular disease and their delivery strategies

**DOI:** 10.1093/rb/rbad103

**Published:** 2023-11-21

**Authors:** Xiaoqian Hong, Geer Tian, Yang Zhu, Tanchen Ren

**Affiliations:** Department of Cardiology of the Second Affiliated Hospital and State Key Laboratory of Transvascular Implantation Devices, Cardiovascular Key Laboratory of Zhejiang Province, Zhejiang University School of Medicine, Hangzhou 310009, China; Department of Cardiology of the Second Affiliated Hospital and State Key Laboratory of Transvascular Implantation Devices, Cardiovascular Key Laboratory of Zhejiang Province, Zhejiang University School of Medicine, Hangzhou 310009, China; Binjiang Institute of Zhejiang University, Hangzhou 310053, China; Binjiang Institute of Zhejiang University, Hangzhou 310053, China; MOE Key Laboratory of Macromolecular Synthesis and Functionalization, Department of Polymer Science and Engineering, Zhejiang University, Hangzhou 310027, China; Department of Cardiology of the Second Affiliated Hospital and State Key Laboratory of Transvascular Implantation Devices, Cardiovascular Key Laboratory of Zhejiang Province, Zhejiang University School of Medicine, Hangzhou 310009, China

**Keywords:** metal ions, cardiovascular disease, tissue engineering, oxidative stress, angiogenesis, ion channel

## Abstract

Metal ions participate in many metabolic processes in the human body, and their homeostasis is crucial for life. In cardiovascular diseases (CVDs), the equilibriums of metal ions are frequently interrupted, which are related to a variety of disturbances of physiological processes leading to abnormal cardiac functions. Exogenous supplement of metal ions has the potential to work as therapeutic strategies for the treatment of CVDs. Compared with other therapeutic drugs, metal ions possess broad availability, good stability and safety and diverse drug delivery strategies. The delivery strategies of metal ions are important to exert their therapeutic effects and reduce the potential toxic side effects for cardiovascular applications, which are also receiving increasing attention. Controllable local delivery strategies for metal ions based on various biomaterials are constantly being designed. In this review, we comprehensively summarized the positive roles of metal ions in the treatment of CVDs from three aspects: protecting cells from oxidative stress, inducing angiogenesis, and adjusting the functions of ion channels. In addition, we introduced the transferability of metal ions in vascular reconstruction and cardiac tissue repair, as well as the currently available engineered strategies for the precise delivery of metal ions, such as integrated with nanoparticles, hydrogels and scaffolds.

## Graphical abstract

**Figure:**
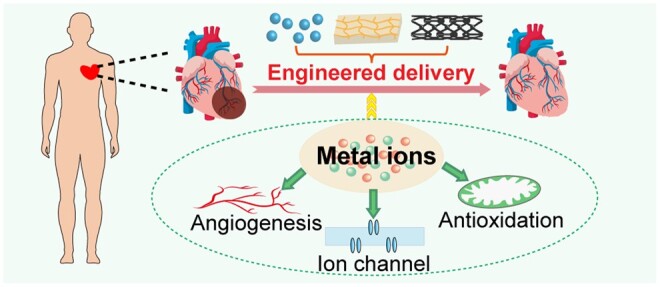

## Introduction

Life activity is the total result of many biologically active substances participating in various chemical reactions, of which metal ions are important parts [1]. Metal ions widely exist in nature. As important parts of all kinds of life forms, they participate in almost all basic biological processes and are crucial to the maintenance of human body’s homeostasis. Effective catalysts formed by metal ions are deeply involved in metabolic reactions [2]. A considerable number of enzymes need metal ions to maintain catalytic activity [3]. Studies have found that more than 50% of modern metabolic pathways depend on metal ions [4]. Take metal ions Mn^2+^ and Zn^2+^ for example, they are important cofactors of many Golgi resident glycosylases. Their homeostasis is necessary for the normal function and stress response of Golgi [5]. Owing to their important roles in tissue homeostasis, exogenous metal ions have the potential to work as therapeutic agents in tissue healing and regeneration. The employment of metal ions in bone tissue engineering has gained increasing attentions [6]; however, their applications in cardiovascular disease (CVD) therapy have remained largely unexplored.

Of the noncommunicable diseases, CVDs, including ischemic heart disease (IHD), stroke, hypertensive heart disease, cardiomyopathy, rheumatic heart disease and atrial fibrillation are now the leading cause of mortality and morbidity worldwide [7, 8]. Strategies aimed at the pathogenesis have been developed to ameliorate the process of CVDs [9]. Taking IHD as an example, after myocardial ischemia occurs, the blood and oxygen supply of the heart are insufficient, leading to abnormal metabolism and excessive production of reactive oxygen species (ROS), accompanied with a large number of cell death [10]. The cellular states of cardiomyocytes are directly related with electrical signal conduction and the activity of ion channel, which are essential for rhythmic contraction of the heart [11]. Protecting cells from the excessive ROS and inflammatory factors [12]; encouraging oxygen and blood vessel restoration [13] and correcting rhythmic contraction functions [14] have gained momentum to ameliorate the disease progression.

Interventional therapy based on medical devices can treat cardiovascular system with focus delivery is the life-saving straw for many types of CVDs. Therapeutic agents such as small molecule drugs [15], stem cells [16, 17] and their derivatives [18], extracellular matrix [19] and growth factors [20] delivered by engineered strategies have shown promising curative effects on CVDs. Among these extrinsic biological active factors, the above-mentioned metal ions possess unique advantages, such as low cost, high stability and safety and diverse drug delivery strategies.

**Figure 1.**
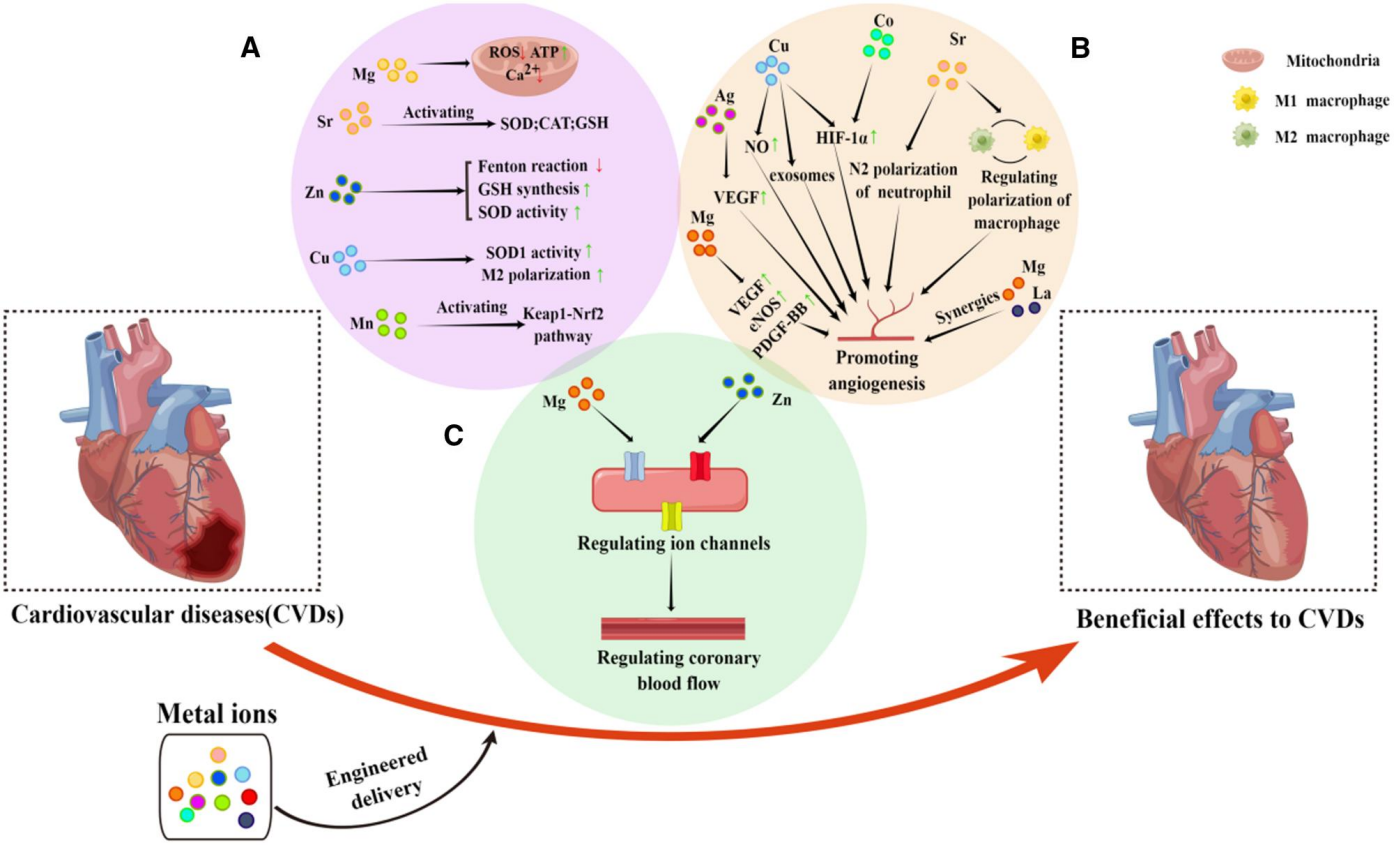
Metal ions treat CVDs through the following mechanisms. (**A**) the antioxid ant mechanism of metal ions. (**B**) The mechanism of promoting angiogenesis by metal ions. (**C**) Metal ions can participate in regulating ion channels.

Here, we provide a brief review specifically on the positive effects of metal ions in the treatment of CVDs from three aspects: (i) protecting cells from oxidative stress, (ii) inducing angiogenesis and (iii) adjusting the effects of ion channels. Moreover, we further discuss the engineering strategies for metal ion delivery which can be used in the treatment of CVDs (Figure 1).

## Positive effects of metallic ions on cardiac vascular disease

### Protecting cells from oxidative stress

Oxidative stress, due to excessive generation of ROS and weak antioxidative defense systems, has been recognized as a molecular trigger of CVDs [12, 21]. In the acute setting of myocardial infarction (MI), excessive ROS generated by abnormal metastasis can significantly lead to mitochondria damage which further lead to cardiomyocyte apoptosis and following inflammatory response [22]. In addition, mitochondrial ROS are also closely related to the development of atherosclerotic plaques. Removal of ROS [23] and inhibition of ROS related genes [24] have been shown to improve myocardial viability, alleviate ischemic injury and the development of atherosclerosis [25]. Taking oxidant stress as the target and reducing ROS production may be an effective treatment strategy for various CVDs [26].

Many metal ions have antioxidant effects both *in vivo* and *in vitro*. Magnesium (Mg) ion is the basic element of mitochondrial function and plays an important role in cell respiration [27]. Mg^2+^ have mild antioxidant properties. Dietary Mg intakes can directly affect the level of oxidative stress in the body, accompanied by changes in inflammatory markers and pro-inflammatory molecules, and affected the resulting myocardial tolerance to ischemia-reperfusion stress [28, 29]. Moderate Mg supplementation has been proven to have a significant effect on protecting DNA from oxidative damage [30]. Liu *et al.* found that Mg^2+^ can be used as a mitochondrial antioxidant to reverse type 2 diabetes-related diastolic dysfunction by increasing ATP, reducing mitochondrial ROS and Ca^2+^ overload and ameliorating oxidative stress [31].

As an essential trace element of the human body, strontium (Sr) can increase the activity of catalase (CAT) and superoxide dismutase (SOD). In the microenvironment of high-level oxidative stress, a certain concentration of Sr^2+^ significantly improved the antioxidant stress capacity of many cells, including leukemia cells of mouse mononuclear macrophage (RAW) [32], which is beneficial for maintaining cellular homeostasis after tissue damage. In addition, Sr-SLA, a dental implant material doped with Sr developed by Zhou *et al.*, can significantly promote bone formation by reducing the level of ROS and increasing the content of glutathione peroxidase (GSH) [33].

Manganese (Mn) ion can also improve the ROS scavenging ability of implants [34, 35]. The Keap1-Nrf2 signaling pathway plays a core role in mediating the adaptive stress response of cells to oxidants [36, 37]. Mn^2+^ can activate the Keap1-Nrf2 pathway as the main antioxidant defense mechanism by upregulating the expression of Nrf2 and promoting its nuclear translocation. At the same time, Mn^2+^ can also inhibit the expression of the endogenous inhibitor Keap1 of Nrf2, increase the antioxidant enzymes CAT and superoxide dismutase 2(SOD2), thereby clearing ROS, inhibiting the activity of osteoclast and regulating cell differentiation.

Zinc (Zn) ion, as a bivalent cation, has no redox activity under physiological conditions but has an activation effect on antioxidant proteins and enzymes [38]. Therefore, Zn^2+^ can be used as an antioxidant to regulate the level of cellular oxidative stress [39, 40]. The relationship between Zn^2+^ and oxidative stress has been studied in cell experiments [41, 42], animal experiments [43] and clinical trials [44]. *In vitro* experiments showed, Zn deficiency increased ROS production in hepatic stellate cells and 3T3 cells [45, 46]. In rodent animal experiments, dietary Zn deficiency also showed an increase in oxidative stress levels of vascular smooth muscle cells *in vivo* [43, 47]. The clinical trial showed that compared with the young subjects, the elderly subjects themselves maintained a low plasma Zn^2+^ level, and after artificial Zn^2+^ supplementation, their plasma lipid peroxidation index significantly decreased [44]. This suggests that the increase of plasma Zn concentration may prevent atherosclerosis through antioxidant stress.

Copper (Cu) is a cofactor of superoxide dismutase 1 (SOD1), which catalyzes the disproportionation of superoxide anion to hydrogen peroxide and oxygen [48]. Although Cu^2+^ cannot directly enhance ROS clearance, studies have shown that a certain concentration of Cu^2+^ release can promote the overall enhancement of the intracellular antioxidant system by upregulating SOD1 and promoting M2 polarization in macrophages [49]. Studies have shown that abnormal copper homeostasis and copper death are closely related to the occurrence and development of CVDs [50].

As the basic elements of many oxidoreductase and complex in mitochondrial respiration, metal ions such as Mg^2+^ help to reduce ROS induced inflammatory and adverse tissue remodeling, while other metal ions such as Mn^2+^ and Zn^2+^ also exhibit certain antioxidant stress effects. Supplementation of the abovementioned metal ions may have beneficial effects on corresponding CVDs.

### Inducing angiogenesis

In biological systems, the transfer of nutrients and wastes in cells and tissues is accomplished through functional vascular networks. Therefore, the maintenance of the vascular system is essential to the health of organisms. During MI, long-term ischemia causes abnormal cardiac metabolism, results in a decrease in cardiac contractile function and ejection capacity, ultimately leads to heart failure [51]. Angiogenesis can increase the blood supply which improves the local oxygen, nutrition, growth factors and cytokine supply of injured tissues [52]. The delivery of therapeutic substances to the ischemic tissue site is an important strategy to promote angiogenesis. For example, growth factors and cytokines [53], relevant cells [54] and their secretome products [55] have been locally or systemically administrated to promote vessel formations in ischemic tissues [56]. Many metal ions are considered to be promising as the supplement to treat ischemic diseases because of their role in promoting angiogenesis.

Cu is an essential cofactor in organisms [57], the lack of Cu can cause deterioration of the tissue function, a recent study involving mice revealed that the Cu level in ischemic heart decreased significantly with the time of MI [58]. Cu^2+^ has been recognized as an effective angiogenesis stimulator, which affects many processes including endothelial cell proliferation, migration and angiogenesis [59–61]. One hypothesis about the angiogenic effect of Cu is mainly through regulating hypoxia-inducible factor-1(HIF-1). Cu participated in multiple steps of HIF-1 regulating target gene expression, such as vascular endothelial growth factor (VEGF) [62, 63]. Another assumption about Cu^2+^ function in pro-angiogenesis is the glutathione peroxidase (GPx)-like function in catalyzing the decomposition of endogenous nitride oxide (NO) donors. NO is the first recognized gas transducer, produced in various biological tissues and an important regulatory factor for key cardiovascular system functions [64–66]. Huang and Yang *et al.* utilized Cu^2+^ to catalyze endogenous NO production, which improve blood compatibility, promote endothelialization and reduce restenosis of Cu-DOTA (1,4,7,10-Tetraazacyclododecane-*N*,*N*′,*N*″,N′″-tetraacetic acid) complex decorated stents [67–70]. Besides, Wang *et al.* introduced copper ions into multilayer coatings via the catechol-Cu coordination to simulate the key functions of healthy endothelial cells, which has the potential to be applied for the modification of blood-contacting devices [71]. Except for the direct functions on endothelial cells, the research of Wang *et al.* found that the exosomes secreted by macrophages stimulated by Cu^2+^ can also upregulate the angiogenesis ability of endothelial cells [72].

At present, the specific mechanism of silver nanoparticles (AgNPs) on wound healing still needs to be further explored. Tian *et al.* found that in a heat injury model, AgNPs treatment induced a higher level of transforming growth factor-β expression at the edge of wounds, which further lead to an increase in VEGF mRNA and keratinocytes migration toward wound area. This might contribute to the function of Ag^+^ released by AgNPs [73].

Mg^2+^ have shown angiogenesis promoting effect by increasing the secretion of angiogenic substances such as VEGF, endothelial nitric oxide synthase (eNOS) [74] and platelet-derived growth factor-BB (PDGF-BB) [75]. Qin *et al.* found that Mg^2+^ upregulated the angiogenesis-related genes HIF-1a and eNOS by activating the Notch signaling pathway in bone marrow mesenchymal stem cells, thereby promoting angiogenesis after implantation [76]. In addition, Mg^2+^ has also been found to promote the formation of new bones and microvessels surrounding implants, which is beneficial for materials-tissue integrations [77–79]. Besides, Zhang *et al.* found that a green tea phenol-Mg^2+^ induced multilayer conversion coating could enhance the adhesion and proliferation of endothelial cells, and improve the biocompatibility of cardiovascular implants [80].

Cobalt (Co) ion is another candidate for angiogenesis stimulation, as it can simulate hypoxia conditions by upregulating HIF-1a, change the expression of hypoxia-related genes involved in angiogenesis and apoptosis, such as VEGF and fibroblast growth factor, stimulate the production of erythropoietin and promote the polarization, migration and homing of endothelial cells [81, 82]. In addition, exosomes secreted by macrophages stimulated by Co^2+^ also have the effect to promote angiogenesis [83]. Research has shown the potential in using a combination of fluoride and Co^2+^ to simultaneously promote osteogenesis and angiogenesis [84].

Sr^2+^ can also act as an accelerator for vascularization by stimulating neutrophils. Compared with pure gelatin scaffolds, Sr-doped gelatin scaffolds stimulated more N2 polarization of neutrophils, accompanied by greater production of angiogenic factors (such as PDGF-BB, VEGF, and stromal cell-derived factor-1). Sr-doped gelatin scaffolds also increased the switch of macrophages to M2 phenotype, ultimately enhanced tissue regeneration [85]. In addition, Mao *et al.* have reported that Sr and Si bioactive ions had synergistic effects on angiogenesis in osteoporotic bone regeneration [86].

Lanthanum (La) is a metallic rare earth element. Luo *et al.* used Mg^2+^ and La^3+^ as biological signal molecules and developed an ion co-delivery system using microspheres as carriers. The results showed that compared with the system that only released Mg^2+^ or La^3+^, the combined system had a more significant effect on angiogenesis, thus promoting the regeneration of vascularized bone tissue [87]. However, further research is needed to explore whether the role of La^3+^ in promoting angiogenesis in cooperation with other metal ions can be applied to the field of CVD treatment.

For most of metal ions the effects in promoting vascularization process are contributed by the upregulated expression of VEGF and other growth factors. Moderate supplementation of the above-mentioned metal ions can have a certain therapeutic effect on the recovery of cardiac function in CVDs characterized by ischemic injury.

### Adjusting the effects of ion channels

Ion channels participate in the physiological function and pathophysiological response of cardiovascular system [88]. For example, calcium channels contribute to atrioventricular conduction and pacemaker activity [89]. In cardiac tissues, K^+^ permeable ion channels have the functions of maintaining cell resting potential, regulating cell tension, cell membrane potential, K^+^ concentration, cell volume and intracellular signal pathways [90]. Given the key roles of ion channels in cardiac conduction and myogenic tension, mutations and dysfunction of these channels can lead to a variety of CVDs and disorders [91]. Pathophysiological conditions characterized by hyperactivity of blood vessels, including hypertension and hyperlipidemia, can lead to changes in the expression or function of coronary artery ion channels [92, 93]. Ion channels, in turn, participate in the regulation of heartbeat and coronary artery blood flow [94–96]. Metal ions are not only the cargo transporting through ion channels, the presence of metal ion also affect the activity of ion channels in turn, which have attracted widespread attention in the cardiovascular field.

Mg^2+^ play an important role in regulating various cation channels in the cardiovascular system. Wang *et al.* found that Mg^2+^ supplementation can alleviate pulmonary hypertension by regulating Mg transporters [97]. The changes in the concentration of Mg^2+^ in myocardial cells or smooth muscle cells may be the basis for altering electrical and mechanical activities of these cells [98]. Mg^2+^ also regulates other cation channels expressed in cells, mainly potassium and calcium channels, which affect the electrical properties of the myocardium and participate in the pathological and physiological processes of CVDs such as arrhythmia. In addition, Mg^2+^ can also regulate myocardial contractility by affecting the intracellular calcium influx [99]. In the cardiovascular field, Mg^2+^ can affect calcium ion homeostasis, vascular tension, peripheral vascular resistance and cardiac output [100].

Zn regulates the activity of multiple ion channels including calcium channel, potassium channel and transient receptor potential channel family of different cell types. Alvarez-Collazo *et al.* studied the effect of extracellular and intracellular Zn^2+^ on L-type calcium current (ICaL) and its modulation by β-adrenergic stimulation in rat ventricular myocytes, and found Zn^2+^ indeed had a regulating effect on transmembrane calcium movement [101]. In addition, Zn^2+^ also induced hyperpolarization of smooth muscle cell membrane by inhibiting the voltage-gated calcium channel of smooth muscle, which resulted in vasorelaxation [102]. These studies show that Zn^2+^ homeostasis may be used as a new target for blood vessel therapy by regulating ion channels.

Metal ions, such as Mg^2+^ and Zn^2+^, play important roles in maintaining ion channel functions and expressions, which can exert positive roles in regulating cardiac conduction and vascular tension. The above-mentioned metal ions can become new targets for the treatment of vascular diseases by regulating ion channels.

Other possible mechanisms can also contribute to the cardiovascular protective effects of metal ions, such as regulating pH [103], modulating the immune system [104], preventing secondary infections in peripheral blood vessels [105] and maintaining vascular elasticity [106]. The rapid accumulation of lactic acid in ischemic myocardium leads to an acidic microenvironment, where weakly alkaline metal ions such as Mg^2+^ and Sr^2+^ may consume acid in the environment and regulate local pH values [107]. In addition, dietary Mg deficiency and Zn deficiency were found to affect the immune system and accelerate the formation of atherosclerosis by exacerbating chronic inflammatory stress [108, 109]. By supplementing the above-mentioned metal ions for immune regulation, it may be possible to reduce arterial inflammation and improve vascular health. Furthermore, molybdenum (Mo) and Mg^2+^ can respectively regulate the plasmalogen and elastic fibers in the vascular wall, which is beneficial for maintaining the elasticity of the arterial wall [106, 110]. For peripheral vascular diseases of diabetes, Cu^2+^, Ag^+^, etc. can prevent the occurrence of local secondary infection in the form of wound dressings [111, 112]. After further research and improvement, the above mechanisms may be utilized for the treatment of CVDs.

## Engineering strategies for metallic ion delivery for cardiovascular disease therapy

**Figure 2.**
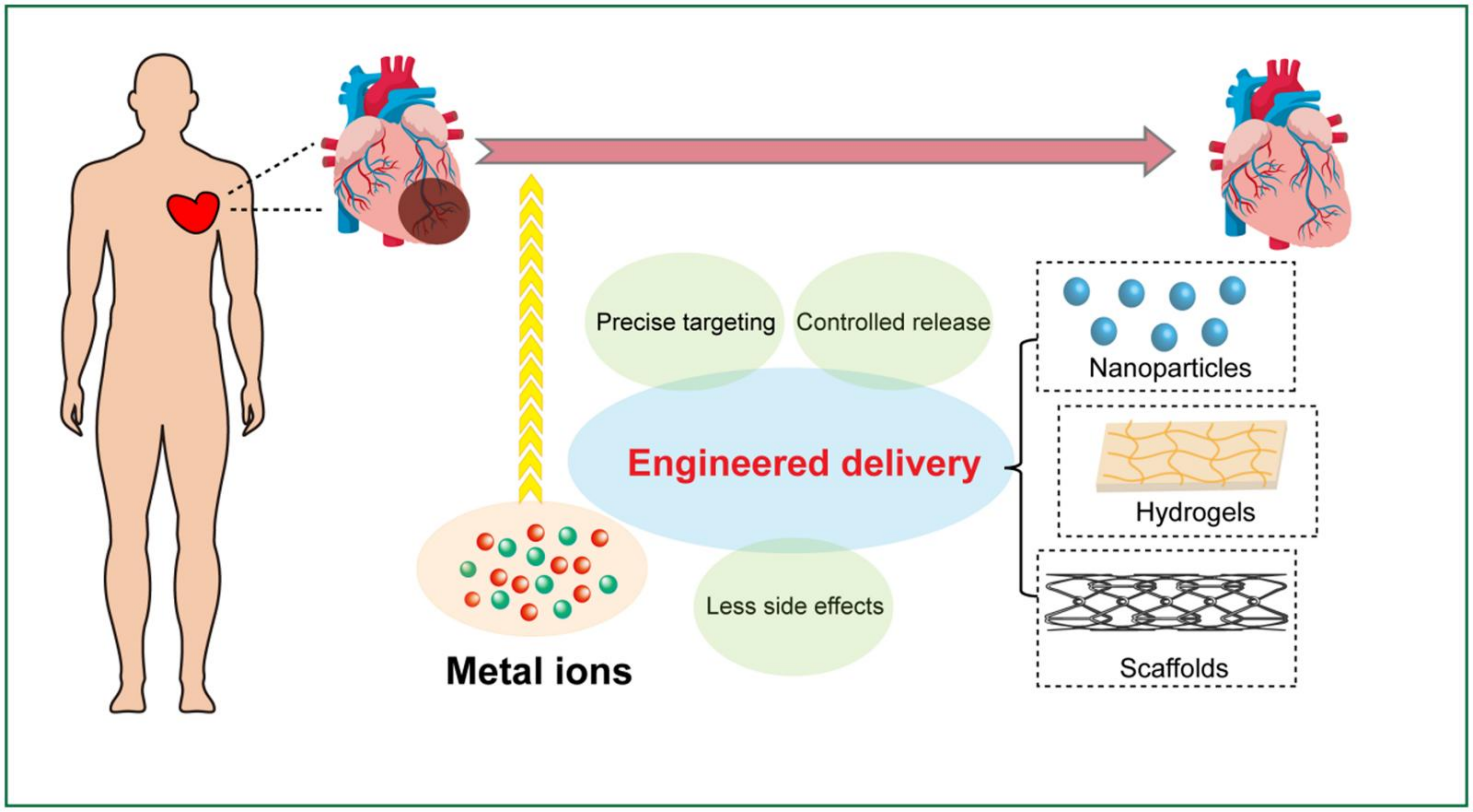
Engineering strategies for metal ion delivery.

Although metal ions have various functions, different tissues and physiological and pathological states have different requisition on the types and doses of metal ions for homeostasis maintenance. Excessive metal ion supplementation has non-negligible side effects, such as affecting enzyme activity, altering cell membrane permeability, increasing hepatorenal burden and damaging the immune and reproductive systems [113]. Taking Mg^2+^ as an example, appropriate local supplementation of Mg^2+^ can effectively promote vascularized bone formation. However, excessive Mg^2+^ entering the circulatory system will cause hypermagnesemia, inhibit the transmission of nerve muscular system to excitement, and cause adverse consequences [114]. Similarly, excessive supplementation of Cu^2+^ is toxic and may lead to neurodegeneration [115]. Dietary reference intake of some metal ions with potential therapeutic effects on CVDs is listed in Table 1 [116, 117]. Therefore, engineering methods for on-demand metal ions delivery can increase the ratio between profits and risks (Figure 2).

**Table 1.** Dietary reference intake of metal ions^a^

Metal ion	Children		Adults	
	**RDA/AI** ^b^	UL	**RDA/AI** ^b^	UL
Cr (mcg/d)	11–15^b^	ND	20–35^b^	ND
Cu (mcg/d)	340–440	1000–3000	900	10 000
Mn (mg/d)	1.2–1.5^b^	2–3	1.8–2.3	11
Zn (mg/d)	3–5	7–12	8–11	40
Fe (mg/d)	7–10	40	8–18	45
Ca (mg/d)	500–800^b^	2500	1000–1200^b^	2500
Mg (mg/d)	80–130	65–110	310–420	350
Mo (mcg/d)	17–22	300–600	45	2000
K (g/d)	3.0–3.8^b^	ND	4.7^b^	ND
V (mg/d)	ND	ND	ND	1.8

a RDA, recommended dietary allowance; AI, adequate intake; UL, upper tolerable level; ND, not determined.

b These values represent the AI.

From nanoparticles and hydrogels to various types of scaffolds and patches [118], the application of innovative biomaterials in the field of therapeutic factor delivery has shed new light on the CVD therapy [119, 120]. In order to transfer metal ions that may have therapeutic effects to the diseased regions, researchers have been introduced a variety of carriers [121].

### Nanoparticles

From a clinical point of view, the system based on nanoparticles is a feasible option to deliver therapeutic drugs to damaged tissues [122, 123]. Nanoparticles can increase targeting and retention of drugs in the desired location of the body, protect unstable molecules from degradation and regulate drug release profile for long-term protection [124]. In addition, the targeted organ delivery ability of nanoparticles can minimize the side effects and toxicity to other organs [125, 126]. Nanoparticles also retain the above advantages as carriers of metal ions. Therapeutic metal ion delivered with nanoparticles and their preparation methods are listed in Table 2.

**Table 2.** Engineering strategies for metal ion delivery based on nanoparticles

Metal ion	Specific biomaterial	Metal ion loading methods	Effects
Zn	LP-loaded ZIF-8 NPs (LP@ZIF-8)	Direct hydrothermal synthesis	Suppress inflammation and regulate lipid metabolism [131]
Cu	The γ-polyglutamic acid and Cu-cosynthesized hydroxyapatite (γ-PGA/CuxHAp)	Ion exchange with Ca^2+^ in hydroxyapatite	Promote angiogenesis [134]
Mn	Ferritin Mn-SOD nanoenzyme (Mito-Fenozyme)	*In situ* synthesis of MnO_2_ into FTn core via Mn^2+^ oxidation	Alleviate oxidative damage [135]
Mn	Mn-contained β-tricalcium phosphate (β-TCP) (Mn-TCP) bioceramics	Co-precipitation method	Scavenge ROS via Nrf2 activation [35]
Zn	Zinc oxide nanoparticles (ZnO-NPs)	Sol-gel method	Reduce oxidative stress and tissue inflammation [136]
Zn	Albumin-based Zn (II)-Quercetin nanoparticles (Que NPs)	Coordination coassembly between bovine serum albumin (BSA) with Quercetin and Zn^2+^	Scavenger active oxygens [137]
Cu	Cu-deposited cerium oxide nanoparticles (CuCe NPs)	Cu^+^ deposition on Ce NPs surface through the mild reduction of Cu^2+^-oleylamine complex.	Promote intracellular antioxidant by upregulating SOD1, promoting M2 polarization of macrophages, increase blood vessel densities [49]
Zn	Green synthesized zinc oxide nanoparticles (GS-ZNPs)	Green chemical method using plant extracts as solvents	Inhibit oxidative stress [138]
Cu	Artificial hybrid nanosized cells (Hynocell)	*In situ* doped Cu nanoclusters into the hollow cavity of FTn	Target ischemic tissues and promote vascular regeneration [133]
Zn, Cu	Zn/Cu dual-doped mesoporous silica nanoparticles (ZC-MSN)	Sol-gel method	Inhibit inflammation and promote angiogenesis [139]

Nanoparticles of metal element or metallic oxide which can decompose *in vivo* are the direct cargoes for metal ions. Created by chemical reduction method or sol-gel method, the size of metal nanoparticles can reach as small as a few nanometers [127], which enable them with nanozyme properties in addition to metal supply. Liu *et al.* developed a subminiature copper-based nanoparticles with simulated enzyme properties for the treatment of ROS-related diseases. Cerium dioxide nanoparticles (Ce NPs) have been widely explored as therapeutic nanoenzymes, which have the characteristics of mimicking SOD and CAT. Im Gwang-Bum *et al.* used Ce NPs as an antioxidant carrier, deposited a large amount of Cu^2+^ on the surface of Ce NPs through the mild reduction of Cu^2+^ oleamine complex and prepared Cu-deposited cerium dioxide nanoparticles (CuCe NPs). As a cofactor of antioxidant enzyme SOD1, Cu can be used in synergistic antioxidant treatment. At the same time, due to its own angiogenic effect, Cu can play a favorable role in the treatment of ischemic vascular disease. Injecting CuCe NPs into the site of MI and local ischemia showed an increase in blood flow perfusion and a reduction in tissue damage [49].

Metal organic framework (MOF) is a three-dimensional (3D) network of nanoparticles containing metal ions originally. In addition, formed by the connection of metal centers and organic groups, the micropores of MOFs enable them to be a potential encapsulation shell and drug carrier for many biomedical applications [128, 129]. Zeolite imidazole skeleton 8 (ZIF-8) is a tetrahedral structural unit formed by connecting Zn^2+^ with N atoms in methyl imidazole ester, and is one of the most promising representatives in MOF [130]. Sheng *et al.* used ZIF-8 as a vehicle to establish a nano losartan potassium (LP)-loaded ZIF-8 drug delivery system. As a zinc-based metal organic skeleton, nanoscale ZIF-8 achieved sustained and stable release of Zn^2+^*in vivo*, which synergistically relieved chronic inflammation and regulated lipid metabolism with LP [131].

Loading metal ions to nanoparticles composed of protein or other polymers by coordination is another important strategy for preparing metal carrying nanoparticles. Polydopamine (PDA) has abundant metal binding sites on its surface, and researchers have utilized this feature to construct Cu^2+^-loaded PDA to achieve local release of Cu^2+^ into the damaged area [132]. Ferritin nanocage (FTn) is a type of recombinant protein assembled from 24 ferritin heavy chain subunits, which compose specific sites to bind with polymetallic ions. Utilizing the metal affinity property of FTn, Zhang *et al.* prepared Cu-containing FTn, fused with cell membrane and coated on PLGA nanoparticles integrated with the secretion from hypoxic stem cells to construct artificial hybrid nanocells that promote vascular regeneration of ischemic tissues [121].

### Hydrogels

Hydrogels, a set of soft materials with large among of water, which have comparable mechanical compliance to native tissues, show unique advantages in tissue engineering [140, 141]. The three-dimensional network structure formed by hydrogels has great capacity in carrying and releasing drugs [142, 143]. When hydrogels are used as the carrier of metal ions, the release profile can be tuned by adjusting the composition, cross-linking degree, pore size, degradation rate and other parameters to meet different treatment requisitions. Therapeutic metal ions delivered by hydrogels and their preparation methods are listed in Table 3.

**Table 3.** Engineering strategies for metal ion delivery based on hydrogels

Metal ion	Specific biomaterial	Metal ion loading methods	Effects
Mg	GelMA/TCS/POSS-Mg hydrogel	Mg-S bond formed by coordination of Mg and TCS	Promote vascularization both *in vivo* and *in vitro* [148]
Zn	Zn_2_ SiO_4_-containing composite hydrogel	Coordination between disulfate modified PEG and Zn^2+^ after physical disperse Zn_2_ SiO_4_ powders into HSA pregel solution	Promote angiogenesis and inhibit oxidative damage [154]
Ag, Co	Polymerized poly(acrylic acid) (PAA) and branched poly(ethylenimine) (PEI) network of Ag nanoparticles decorated polypyrrole nanotubes (AgPPy) and Co ions (PPCA hydrogel)	Electrostatic interaction between metal ion and PAA and branched PEI network	Promote notable inflammatory reduction and prominent angiogenesis regeneration [155]
Ag, Cu	Chitosan (CS)-Ag-Cu hydrogel	Chelation between -NH_2_/-OH and metal ion	Promote cell migration and angiogenesis [156]
Mg	MgO/MgCO_3_@poly(lactide-co-glycolide)(PLGA) hydrogel (PMM hydrogel)	Physical disperse MgO and MgCO_3_ particles into the prepared PLGA hydrogel	Stimulate cell migration, promote cell adhesion and proliferation [152]
Ag	AgNPs-loaded dopamine-grafted hyaluronic acid (HA-DA)/3-aminophenylboronic acid-grafted oxidized methyl cellulose (OMC-PBA) hydrogel	Coordination between dopamine and Ag^+^ after disperse AgNPs into the hydrogel precursors	Promote antioxidant stress, antibacterial, cell proliferation, anti-inflammatory and angiogenesis [157]
Sr	SrCO_3_/human serum albumin (HSA) composite hydrogel	Coordination between disulfate-modified PEG and Sr^2+^ after physical disperse SrCO_3_ into pregel solution	Reduce cardiomyocyte apoptosis and increase angiogenesis [153]
Mg	GelMA-BP-Mg microspheres	Coordination of metal ion and BP	Promote vascularization [149]
Mg	Poly (hydroxypropyl acrylate-co-acrylic acid)-Mg^2+^ hydrogel (poly (HPA-co-AA)-Mg^2+^)	Coordination of metal ion and carboxylate	Promote the formation of new blood vessels, the proliferation and migration of fibroblasts and M2 polarization of macrophages [150]

Based on metal-ligand coordination chemistry, metal ions can directly bind to various chelating ligands coupled on the backbone of hydrogel polymers [144, 145]. The metal ions coordinated in hydrogel network can work as the ionic crosslinker to mold or reinforce the hydrogels. Ligands such as alginates, bisphosphate (BP), catechol, thiolate, which have shown high associate constants to many polyvalent metal ions, have been extensively studied for ion loading. Due to the dynamic property of ionic crosslinking, ion release can be triggered by ion exchange in physiologic environment. The variation of pH, ionic strength, redox state *in vivo*, the present of ions with higher associate constant, the degradation of hydrogel polymer have been utilized to design hydrogels with programable ion release [146, 147]. Zhang *et al.* prepared Mg^2+^-binding double crosslinked hydrogel through the addition of thiolated chitosan (TCS) in gelatin methacryloyl (GelMA) and polyhedral oligomeric silsesquioxane (POSS) photocrosslinked hydrogel. Hydrogel showed sustained release of Mg^2+^ for more than 15 days, which promoted angiogenesis *in vitro* and *in vivo* [148].

Hydrogel can be prepared into microspheres (microgel) for better shape adaptability and easier minimal invasive introduction. Zhao *et al.* obtained BP grafted GelMA microspheres by microfluidic method and loaded Mg^2+^ through coordination between BP and metal ion. GelMA-BP microspheres could be injected by syringe and achieved sustained local release of Mg^2+^ to promote local endothelial cell growth [149]. Cui *et al.* used the coordination between metal ions and carboxylate to introduce Mg^2+^ into the microgel rich in carboxyl groups. Microgel was spread and covered the entire damaged part seamlessly and promote tissue regeneration [150].

In addition to introducing metal ions through direct coordination with hydrogel polymer, metal-loaded nanoparticles can also be introduced into hydrogels as an alternative metal ion source [151]. By embedding metal nanoparticles in the hydrogel matrix, the release of metal ions could be further stabilized. Moreover, hydrogel with *in situ* gelation property could be used to load metal ion carrying nanoparticles, which further increase the compliance and injectability of the implants. Zhou *et al.* used PLGA/1-methyl-2-pyrrolidinone solution, which is an FDA-approved *in situ* forming implant (ISFI), to load MgO and MgCO_3_ particles as alternative Mg^2+^ sources. This ISFI could completely fill the irregular defects *in vivo*. As MgO and MgCO_3_ had different degradation rates, the sustainable and stable release of Mg^2+^ was achieved by adjusting the weight ratio, the two particles doped in the hydrogel [152]. Other than the simple physically dispersion, Chang *et al.* used disulfate-modified PEG to coordinate Sr^2+^ in SrCO_3_ nanoparticles, which also act as a crosslinker for hydrogel. The dynamic binding enabled minimal invasive injection into myocardial tissue of the hydrogel. After I/R, the Sr^2+^-containing hydrogels were injected into murine infarcted myocardium, and the researchers observed the reduction of cardiomyocyte apoptosis and the increase of angiogenesis. This indicates that locally released Sr^2+^ has a cardioprotective effect against I/R injury [153].

### Solid scaffolds

Solid scaffolds with certain mechanical strength are widely used for tissue engineering to support the configuration of injured tissues [158]. Scaffolds has been designed into different modality. In the case of CVDs, intravascular stents which can provide support for the arterial wall and ensure blood supply, have become a routine treatment for thrombus [159, 160]. Cardiac patches, which can provide necessary mechanical support for damaged myocardium, have been reported to significantly improve cardiac function [161]. Other than mechanical support, scaffolds also provide niches for cell growth and drug reservoirs, metal ions are one of the popular therapeutic factors that used for scaffold designed. We listed the therapeutic metal ion delivered by scaffolds and their preparation methods in Table 4.

**Table 4.** Engineering strategies for metal ion delivery based on scaffolds

Metal ion	Specific biomaterial	Metal ion loading methods	Effects
Zn, Mg, Cu	Zn-Mg-Cu alloy	Alloy smelting	Augment immunoregulation, angiogenesis, and anti-infective activity [162]
Mg	PLGA/oligolactide-grafted Mg(OH)_2_ (RA-Mg-OLA) scaffold	Ultrasonic nanocoating method	Reduce inflammatory response [167]
Mg, Sr	3D-printed Mg-/Sr-doped Ca silicate scaffold	Blending and sintering	Promote the angiogenesis behavior [168]
Zn	Zn-loaded β-tricalcium phosphate/poly(l-lactic acid) (TCP/PLLA) scaffold	Wet precipitation and blending	Direct stem cell fate and trigger a pro-healing immune stimul [169]
Co	Co-doped Ca_10_Li(PO_4_)_7_ (CLP) scaffold	Substitution and sintering	Enhance angiogenic property [170]
Ca, Mg	Ca Mg silicate (CMS)/graphene oxide (GO)/silk fibroin (SF) composite scaffold	Chemical precipitation, blending and lyophilization	Upregulate angiogenesis genes and promote angiogenesis of human umbilical vein endothelial cell [171]
Mg	Mg-enriched graphene oxide nanoscroll (MgNPs@GNS) deposited decellularized bone matrix scaffold	Electrostatic interactions with GO surface groups and then reduced under solvothermal conditions	Stimulates angiogenesis [78]
Sr	Tannic acid (TA)/Sr^2+^-coated silk/graphene oxide-based meniscus scaffold	Coordination with phenolic hydroxyl groups of TA	Eliminate ROS, promote cell migration and facilitate ECM secretion [172]
Cu	Catechol-mediated and Cu-incorporated multilayer coating modified SS wires	Catechol-mediated layer-by-layer multilayer coatings via catechol-Cu coordination	Generate NO *in situ* [71]
Cu	Epigallocatechin-3-gallate-Cu@rapamycin/bivalirudin-modified PLLA stents (EGCG-Cu@Rapa/BVLD-modified PLLA stents)	Catechol-Cu coordination	Provide sustained NO release, prevent restenosis and promote endothelial healing after stenting [173]
Cu	CuII-dopamine (DA)/hexamethylenediamine (HD) coating modified 316 L SS vascular stents	Catechol-Cu coordination	Catalyze NO release, improve the antithrombogenicity, re-endothelialization and further anti-restenosis [165]
Cu	Stents grafted/loaded with catechol groups/Cu^2+^ films	Catechol-Cu coordination	Catalyze NO release *in situ* [166]
Cu	Fibroin/chitosan (SF/CS)/Cu coating modified stents	Metal-protein coordination	Regulate NO catalyst generation [174]
Cu, Ag	PH-responsive silk fibroin-based CuO/Ag micro/nano coating polyetheretherketone (PEEK)	Particles were incorporated into the porous surface of PEEK through PDA and silk fibroin layers	Promote antibacterial ability and angiogenesis [175]
Cu	CuS@electrospun nanofiber (ENF) composite (ENFC)	Polyelectrolyte complexation and genipin-involved cross-linking reaction	Promote the capillary tube formation of endothelial cells [176]
Zn	Zn^2+^ cross-linked quaternized cellulose (QC)-sodium alginate (SA) composite sponges	Crosslink QC and SA with Zn^2+^	Promote epithelial regeneration and mitigate inflammatory cell infiltration [177]

Alloy stents can serve as the carriers for metal ions inherently. For bioabsorbable stents, the metal ions released during degradation can exert therapeutic effects locally. Zhao *et al.* prepared a new type of degradable Zn-Mg-Cu alloy scaffold by integrated additive manufacturing. The Zn alloy showed moderate degradation rate. Mg enhanced mechanical strength of Zn alloy, and the released Mg^2+^ showed immunoregulation effects. Cu^2+^ released during degradation enhanced angiogenesis and anti-inflammation function of the scaffold, which promoted tissue regeneration [162].

Polymer scaffolds are extensively studied for tissue engineering. Although the adding of metal ions in polymer is not as straightforward as doping in alloy, many studies have been proceeded to add metal ions or metal-related nanoparticles in organic scaffolds. Wang *et al.* constructed a composite scaffold composed of piezoelectric whitlockite and poly(ε-caprolactone) (PCL) through 3D printing technology. Whitlockite is a natural Mg containing Ca phosphate that has sustained release of Mg^2+^ and Ca^2+^ [163]. Besides, Zhang *et al.* synthesized uniform Zn silicate nanoparticles with spindle-shaped morphology using hydrothermal method and incorporated them into PCL electrospun nanofibers, obtaining a bioactive nanofiber scaffold loaded with Zn^2+^. The continuous release of Zn and silicon ions showed beneficial effects in stimulating vascular regeneration in both *in vivo* and *in vitro* experiments [164].

Surface coating technology can increase biocompatibility of biomaterials without affecting the structure and mechanical properties of the scaffolds, it is especially important in improving hemocompatibility for a series of blood-contacting materials. The surface coating of certain metal ions can increase the endothelialization (i.e. Mg^2+^ [80]) and anti-thrombus effects (i.e. Cu^2+^). One of the most commonly used metal ion functionalization method is the metal-phenol network coating, which is formed by coordinating various polyphenols with metal cations [165, 166]. This strategy is broadly applicable due to its high biocompatibility and high stability.

### Others

Besides nanoparticles, hydrogels and scaffolds, many other methods for metal ions delivery are constantly being developed, which are listed in Table 5.

**Table 5.** Engineering strategies for metal ion delivery based on others

Metal ion	Specific biomaterial	Metal ion loading methods	Effects
Sr, Mg	PLGA micro-cage-like structures loaded with Sr- and Mg-doped hydroxyapatite (HA) (Sr/Mg@HA/PLGA-CAS)	Ion exchange of Sr^2+^/Mg^2+^ with Ca^2+^ in hydroxyapatite	Assist angiogenesis [181]
Zn	Zn-MOF encapsulated degradable MNs array	Encapsulate ZIF-8 into a photo-crosslinked methacrylated hyaluronic acid (MeHA) through the molding method	Accelerate epithelial regeneration and neovascularization [178]
Ag, Ca, Zn, Cu	Freeze-thawing CS/ions hydrogel coated gauzes	Freeze-thawing method	Promote granulation formation, collagen deposition and maturation, re-epithelization, angiogenesis and inhibit inflammation [182]
Cu	Cu-EGCG capsules	Layer-by-layer assembly through the coordination of EGCG with Cu^2+^	Induce the secretion of VEGF, promote angiogenesis and restore local blood supply [180]
Cu	Cu-albumin microbubble	The coordination between N-terminal tripeptide chains and Cu^2+^	Restore blood vessel density and improve cardiac contractility [179, 183]

Microneedles (MNs) is a typical minimally invasive local drug delivery method. Compared with ordinary patches, drugs can penetrate into the matrix of the target location much more easily; compared with direct injection, MNs are more painless and MNs can act as drug reservoirs for sustained release. Yao *et al.* combined MOF and MNs and designed a Zn-MOF-encapsulated degradable MNs array. This MNs array can effectively release Zn^2+^ and has been found to significantly accelerate epithelial regeneration and neovascularization [178]. However, the use of MNs array for metal ion delivery in CVD therapy has not been widely reported.

Other materials with micron level size have also shown advantages in the delivery of metal ions, because their dimensions are suitable for minimally invasive implantation, and their capacity in loading and releasing metal release are easy to tune. For example, micron sized Cu-albumin microbubbles were prepared for ultrasound-guided organ specific delivery of Cu. The local release of Cu at the ischemic myocardium, ultimately restoring local vascular density and improving cardiac contractility in rhesus monkeys [179]. Duan *et al.* synthesized micron level metal polyphenol capsules (Cu-EGCG capsules) by coordinating Epigallocatechin-3-gallate (EGCG) with Cu^2+^. The continuous release of Cu^2+^ induce VEGF secretion, promote angiogenesis and restore local blood supply in hindlimb ischemia models [180].

As the development of control release materials, more methods with their own characteristics are being developed. The criteria of the strategies will be the translational applications for the treatment of specific CVDs, which is conducive to further improving the treatment effect.

## Conclusion and future prospective

Metal ions have shown positive roles in inhibiting oxidative stress, promoting vascular regeneration and regulating ion channels. They also have unique advantages in therapeutic application, such as broad resource, high stability and safety and diverse drug delivery methods. More and more studies have begun to focus on the clinical transformation of metal ions in regenerative medicine and tissue engineering, especially in the cardiovascular field. Strategies for the controlled local delivery of metal ions based on various biomaterials are being designed and have received more and more attentions. Although the current application of metal ion delivery is still focused on skin damage repair and bone repair, with the continuous exploration and discovery of the importance of metal ions in the cardiovascular field, we believe that in the near future, more and more diverse engineering strategies will be applied to the treatment of CVDs.

The delivery strategies of metal ions for biomedical applications are various, most of which use nanoparticles, hydrogels and solid scaffolds as carriers. Different carriers have different application ranges and corresponding advantages and disadvantages. Nanoparticles are widely used in medical imaging, gene and drug delivery, pathogen and protein detection, and tissue engineering [184, 185]. They have good encapsulation performance and can achieve precise treatment through active/passive targeting [186]. However, there is a relationship between the size and toxicity of therapeutic nanoparticles, and smaller nanoparticles tend to aggregate, leading to poor biological distribution [187, 188]. Hydrogels are widely explored and applied in cosmetic medicine, wound dressings, drug delivery, disease models, tissue repair and regeneration and other fields [189]. They have good biocompatibility and biodegradability, and have the ability to support cell interactions and tissue volume [190]. Nevertheless, their hydrophilicity may lead to low encapsulation efficiency of hydrophobic bioactive molecules and drugs, and their highly porous structure may lead to cargo leakage during transportation [191]. Solid scaffolds play an important role in cartilage repair, bone tissue engineering, heart repair and drug delivery [192]. They have good mechanical strength that the aforementioned carriers do not possess, and their interactions with cells are also conducive to stimulating the formation of functional tissues [193, 194]. However, the application of scaffolds is still limited by their immunogenicity, and due to their significant differences in mechanical and biochemical properties from natural tissues, it is difficult to simulate the microenvironment inside the body well [195]. Therefore, when choosing metal ion delivery strategies, their characteristics should be fully considered to achieve safer and more efficient drug delivery.

At present, the supplementation of metal ions is still mainly based on oral foods with relatively high levels, and clinical research on the treatment of CVDs through local delivery of metal ions is still lacking. The current clinical research mainly focuses on the potential therapeutic effects of metal ion concentration changes caused by cyclic drug administration on CVDs. Woods *et al.* conducted a randomized, double-blind controlled study on 2316 suspected acute MI patients and found that receiving intravenous magnesium sulfate significantly reduced early mortality and left ventricular failure rates. The efficacy of magnesium sulfate in reducing early mortality after MI might be attributed to the beneficial cardiovascular effects of Mg^2+^ at pharmacological concentrations [196]. In addition, clinical studies have also found a beneficial trend of Mg^2+^ in reducing the rate of restenosis after percutaneous coronary angioplasty [197].

To facilitate translational application of metal ion as therapeutic agent for CVD, the release of metal ion is an important issue. First, due to the varying sensitivity of different tissues to different metal ions, and the difficulty in determining the types and dosages of metal ions that need to be supplemented for different degrees of injury, it is difficult to accurately delineate the concentration range of metal ions that are effective in treating body injuries and have no obvious toxic side effects. In addition, the increase in metal ion concentration caused by the release of local metal ions has certain limitations for combined medication. This is because metal ions can not only form insoluble complexes with drugs, affecting their efficacy, but also react with drugs, affecting their stability. Finally, most metal ions are excreted through organs such as the liver and kidney, making it difficult to determine the potential toxicity of long-term treatment to these organs. Therefore, further research is needed to continuously improve the accuracy of on-demand delivery of metal ions.

The important roles and corresponding mechanisms of many metal ions, such as Cu^2+^, in metabolism and immune regulation are constantly being explored. The emergence of various new technologies and methods is very beneficial for the study of the mechanism of metal ions acting *in vivo*. AlphaFold can utilize deep learning techniques to predict the interactions between metal ions and major proteins through molecular docking simulations [198]. In addition, the localization and movement of metal ions within cells can be tracked through artificial intelligence (AI) comparison [199], fluorescence probes [200] or electron microscopy [201]. Moreover, the metabolic process of metal ions in the body can also be tracked through isotopes [202]. These technical means can be applied to further exploration of the role of metal ions in CVDs, in order to further determine the types and dosage ranges of metal ions that need to be supplemented for specific CVDs. When the time is ripe, combining the characteristics and advantages of different transmission strategies, local transmission of metal ions may achieve more effective treatment of CVDs.

In this article, we summarize the beneficial effects of different metal ions on the therapy of CVDs. In addition, we focus on the transferability of metal ions in vascular reconstruction and cardiac tissue repair, as well as the corresponding clinical transformation application design at present. Although further researches are still needed to explore the specific mechanisms of metal ions on cardiovascular functions and increase the precision of on-demand delivery before translation, the clinical application prospects of metal ions are still expected.
